# Inferior labrum tears can accompany SLAP lesions and inferior labrum repair with SLAP lesion treatment results in satisfactory clinical outcomes at a minimum 2-year follow-up

**DOI:** 10.1007/s00402-025-05940-7

**Published:** 2025-06-02

**Authors:** Ethem Burak Oklaz, Asim Ahmadov, Furkan Aral, Mustafa Can Erdem, Inci Hazal Ayas, Ulunay Kanatli

**Affiliations:** Gazi University, Ankara, Turkey

**Keywords:** Inferior labrum tears, SLAP lesions, Traction, Injury, Clinical outcomes

## Abstract

**Introduction:**

The present study aimed to determine the prevalence of concomitant inferior labrum tears in patients with superior labrum anterior posterior (SLAP) lesions, and evaluate the clinical outcomes of arthroscopic inferior labrum repair performed concurrently with SLAP lesion management.

**Materials and methods:**

This retrospective case series was conducted on patients who underwent shoulder arthroscopy for SLAP lesions between 2017 and 2022. The study group included patients who had SLAP lesion treatment (tenotomy, tenodesis, or repair) and inferior labrum tear repair, with a minimum follow-up of 24 months. Demographic data and clinical characteristics of the patients were assessed. Outcomes were evaluated using the Oxford Shoulder Score (OSS), Subjective Shoulder Value (SSV), and Visual Analogue Scale (VAS). Furthermore, the proportion of patients who met the Minimal Clinically Important Difference (MCID), Substantial Clinical Benefit (SCB), and Patient Acceptable Symptom State (PASS) thresholds for the patient-reported outcome measures (PROMs) were determined.

**Results:**

A concomitant inferior labrum tear was identified in 11% (*n* = 32) of 278 patients who underwent surgery for SLAP lesions. Among these, 26 patients (mean age, 43.6 ± 9.8 years; 50% male/female; 77% dominant extremity involvement; mean follow up, 46.8 ± 20.5 months) who met inclusion criteria were included in the study. 17 (65%) patients had a history of sudden arm traction during heavy lifting. Significant improvements in PROMs were observed at the final follow-up (*p* <.001 for all scores). The rates of patients achieving MCID, PASS, and SCB were determined, respectively, OSS (96%, 81%, 84%), SSV (100%, 77%, 84%), and VAS (88%, 81%, 81%).

**Conclusions:**

Inferior labrum tears are a pathological condition that may accompany SLAP lesions, and patients with both lesions usually have a history of traction-related injuries. In these cases, successful clinical outcomes could be achieved through patient-specific management of the SLAP lesion and repair of the inferior labrum.

**Level of evidence:**

Level IV, therapeutic study, retrospective case series.

## Introduction

Superior labrum anterior posterior (SLAP) lesions are tears of the glenoid labrum at the 11–1 o’clock position, identified in 4–26% of shoulder arthroscopies [1–6]. SLAP lesions are associated with several injury mechanisms, including falling onto a shoulder or outstretched arm, and forceful traction applied to the upper extremity [7–9]. A sudden pulling force on an arm is an important mechanism in traction-related injuries. Traction load on the arm, particularly when lifting heavy objects, generates an inferior translational force within the glenohumeral joint. These forces increase tension on the long head of the biceps tendon (LHBT), potentially leading to SLAP lesion [3, 7, 8].

Remarkably, inferior translational forces within the glenohumeral joint have also been defined as a potential leading factor for inferior labrum tears [10]. These tears at the 5 to 7 o’clock position, opposite to the typical location of SLAP lesions, constitute approximately 10% of glenoid labrum injuries and are termed ‘inferior labrum anterior-posterior (ILAP) lesions’ following the same naming convention [10–13]. Although similar injury mechanism suggests a potential relationship between superior and inferior labrum tears, studies examining labral pathologies associated with SLAP lesions have typically considered inferior labrum tears as an extension of anterior or posterior labral tears, and limited information is available regarding this injury configuration [14–17]. Moreover, in our clinical practice, concomitant inferior labrum tears have been observed in some cases during the arthroscopic management of SLAP lesions. All of these findings prompted us to question whether inferior labral injury could be a pathology associated with SLAP lesions. A clearer understanding of this relationship could enhance the current knowledge of labrum pathologies, potentially enabling orthopedic surgeons to recognize more specific injury patterns.

Therefore, the present study has two primary objectives: (1) to determine the prevalence of concomitant inferior labrum tears in patients with SLAP lesions, and (2) to evaluate the clinical outcomes of arthroscopic inferior labrum repair performed concurrently with SLAP lesion management in these patients. We hypothesized that patients with SLAP lesions, particularly those caused by forceful traction, may have concomitant inferior labrum tears, and that in these patients, appropriate SLAP treatment combined with inferior labrum repair could provide satisfactory clinical outcomes.

## Methods

**Fig. 1:**
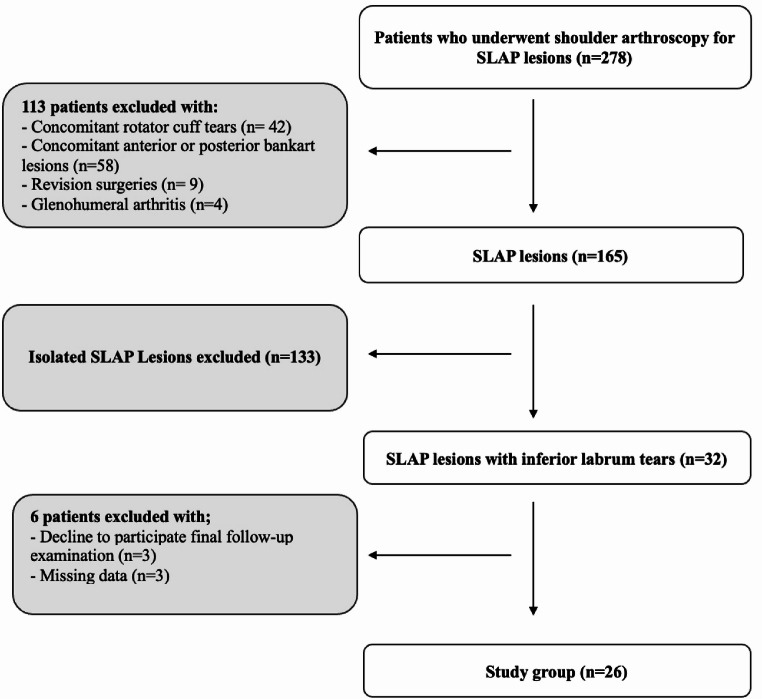
Flowchart of the patient selection process

This study was designed as a retrospective cohort study analyzing patients diagnosed with SLAP lesions who underwent arthroscopic surgery performed by a single senior surgeon (U.K.) between February 2017 and October 2022. Institutional review board approval was received from Gazi University Ethics Committee (Protocol: 2024-656). The data of 278 patients were collected. Patients with concomitant rotator cuff pathologies, concomitant anterior or posterior labrum tears, isolated SLAP lesions, revision surgeries, glenohumeral osteoarthritis, decline to participate final follow-up, and missing data (preoperative history and examination informations) were excluded from the study. The study group was formed patients with SLAP lesions and concomitant inferior labrum tears. The patient selection process is described in the flowchart (Fig. 1).

### Surgical technique and postoperative rehabilitation

The patients were operated on in the semilateral decubitus position. All surgical procedures were performed with the patient under an interscalene block with or without general anesthesia. An arthroscope was inserted through the posterior portal to assess the anterior and superior labrum. The arthroscope was then placed in the anterosuperior portal to assess the inferior and posterior labrum. Subsequent to the determination of SLAP lesion and inferior labrum tear, the initial procedure involved repair of the inferior labrum using minimum two double-loaded suture anchors (2.9 mm GRYPHON, DePuy Mitek, Raynham, MA, USA). The first anchor was placed posterior to the inferior glenoid, creating an accessory portal at the 7 o’clock position. Subsequently, additional anchors were placed sequentially, at intervals of 8–10 mm. The feasibility of placing anchors in the anterior aspect of the inferior glenoid through the existing portals was assessed. If these portals were deemed inadequate, a trans-subscapularis portal was created at the 5 o’clock position to facilitate anchor placement. A simple vertical suture and horizontal mattress suture were performed in each anchor to ensure optimal stability. Labral repair was considered complete when the entire labrum was securely reattached to the glenoid rim.

Patients with SLAP lesions were first assessed for any biceps tendon pathology. In cases where no LHBT pathology was present, factors such as younger patient age, and expectation of a rapid return to sporting activities typically led to the selection of SLAP repair [7, 14, 18, 19]. On the other hand, in older patients who prioritize pain relief and a shorter rehabilitation period over high functional demands and have no concerns about cosmetic deformity, biceps tenotomy was generally preferred [20, 21]. In cases with SLAP lesions associated with biceps pathologies, SLAP repair was not performed; instead, tenodesis or tenotomy was selected based on the patients’ preoperative expectations [21–25].

After the surgery, patients underwent the postoperative rehabilitation program. The affected arm was kept in a sling for 6 weeks after surgery. Passive shoulder flexion, abduction, internal and external rotations, and isometric shoulder strengthening exercises were introduced after the first week and continued until the end of the sixth week. Active-assisted shoulder range of motion exercises were introduced between weeks six and ten. The rehabilitation protocol progressed with active shoulder joint exercises and stretching exercises beginning after the tenth week, while resistance strengthening exercises commenced after the twelfth week. Full unrestricted return to daily activities was permitted after six months, and full unrestricted return to sports was allowed between the ninth and twelfth months.

### Data collection

An author (A.A.), who was blinded to the details of the current study, conducted a retrospective review of the patient charts documented by the orthopedic surgery department. The demographic and clinical data of the patients, including age, sex, affected side, dominant side, physical work intensity, mechanism of injury, preoperative pain and functional scores were obtained from these medical records. Patients were subsequently contacted and invited for a final follow-up evaluation. This evaluation was conducted by another author (I.H.A.) who was blinded to the details of the current study. Pain levels were assessed using the Visual Analog Scale (VAS), whereas shoulder function was evaluated using the Oxford Shoulder Score (OSS) and Subjective Shoulder Value (SSV). At the same time, a satisfaction questionnaire with an anchor question was administered to calculate the Minimal Clinically Important Difference (MCID), Substantial Clinical Benefit (SCB), and Patient Acceptable Symptom State (PASS) for patient-reported outcome measures (PROMs).

### Statistical analysis

Statistical analyses were performed using SPSS for Mac (version 28.0; IBM Corp.). Descriptive statistics and frequency distributions were calculated for all the variables. The Shapiro-Wilk test was used to assess the distribution of continuous variables. To compare preoperative and postoperative PROMs the Wilcoxon signed-rank test was applied. Clinically significant thresholds were established by calculating the MCID, SCB, and PASS values for OSS, SSV, and VAS, using both anchor-based and distribution-based methodologies. Given that a significant proportion of patients experience improvements in clinical outcomes, MCID for PROMs was calculated using a distribution-based method, applying half the standard deviation of the overall change in each outcome measure across the entire cohort [26–28]. Patients were considered to have met the MCID if their improvement in the outcome score exceeded a predefined threshold. The PASS was assessed using an anchor-based method. At the final follow-up, patients were asked “Considering your daily activities, including both pain and function, are you overall satisfied with the outcome of the surgery?”. Receiver operating characteristic (ROC) curve analysis was used to determine threshold values for PROMs (based on changes in scores) using dichotomous (yes/no) responses to this question. Patients whose score changes exceeded these thresholds were considered to have met the PASS. Assessment of SCB was also conducted through an anchor-based approach. To assess changes in pain levels, the patients were asked, “Has there been any change in your pain following surgery?”. To evaluate functional improvement, they were asked, “Has there been any change in your functional or physical capacity since the surgery?”. A 15-point scale was used for responses to both questions, with answers ranging from 0 (indicating a very great deal worse) to 15 (indicating a very great deal better) [29]. For the SCB calculation, scores between 7 and 9 were classified as the no improvement group, scores between 10 and 12 as the minimal improvement group, and scores between 13 and 15 as the substantial improvement group [29]. Differences between the ‘no improvement’ and ‘substantial improvement’ groups were analyzed to calculate SCB using ROC curve analysis [28–30]. The SCB for the VAS score was calculated using the anchor question on pain, whereas the SCB for the OSS and SSV scores was determined using the anchor question for function [30]. ROC analysis was then used to assess score changes and determine the corresponding SCB thresholds. Patients whose score changes exceeded these thresholds were considered to have met the SCB. In the ROC curve analysis, an area under the curve (AUC) greater than 0.7 was considered acceptable, while an AUC exceeding 0.8 indicated excellent predictive ability [31].

## Results

**Fig. 2:**
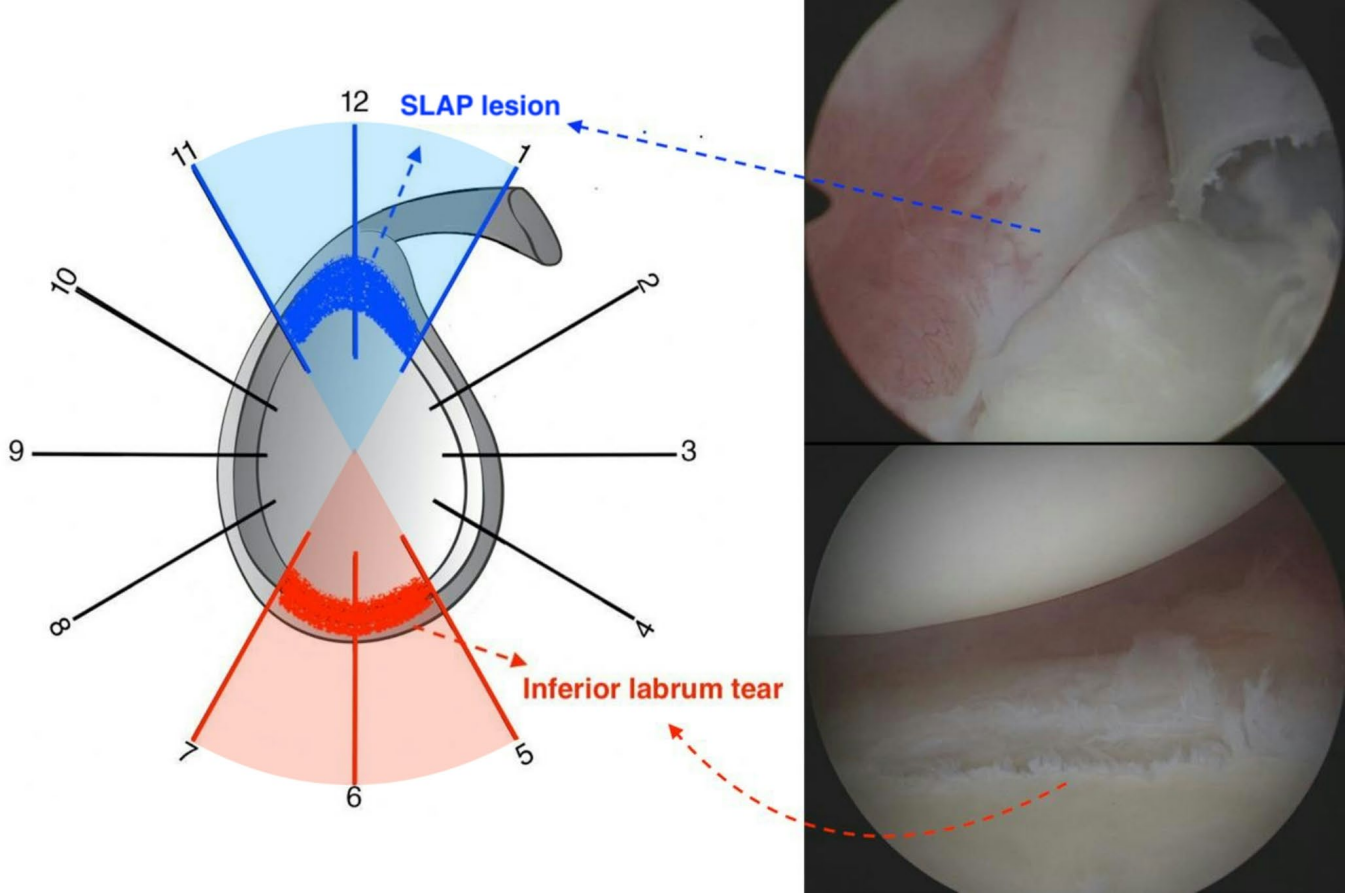
Illustration of inferior labrum tear and SLAP lesion

A concomitant inferior labrum tear was identified in 11% (*n* = 32) of 278 patients who underwent surgery for SLAP lesions (Fig. 2). Of these 32 patients, 26 with complete data and final follow-up participation were included in the study. The mean age of the patients was 43.6 ± 9.8 years (range, 28–62 years). Male and female participants were equally represented in the study population (Table 1). Half of the patients worked in light, 31% in intermediate, and 19% in heavy occupations. The average follow-up duration was 46.8 ± 20.5 months (range, 24–94 months). The dominant extremity was affected in most of the patients (*n* = 20, 77%). Sudden traction of the arm while lifting a heavy object was the most common mechanism of injury (*n* = 17, 65%). Other mechanisms included falling onto an outstretched arm (*n* = 3, 11%), overhead or throwing sports (*n* = 2, 8%), falling onto shoulder (*n* = 2, 8%), and insidious onset (*n* = 2, 8%). All the patients in the study group had type 2 SLAP lesions. In the management of SLAP lesions, tenodesis was performed in 11 (42%) patients, repair in 9 (35%), and tenotomy in 6 (23%).

**Table 1:** Patient characteristics

Variable	*n* (%) or mean ± SD (min-max)
**Age**,** yr**	43.6 ± 9.8 (28–62)
**Sex (M/F)**	13 / 13 (50% / 50%)
**Affected side (R/L)**	16 / 10 (62% / 38%)
**Dominance**	
Dominant arm	20 (77%)
Non-dominant arm	6 (23%)
**Symptom duration**,** mo**	34.6 ± 20.2 (6–72)
**Mechanism of injury**	
Insidious onset	2 (8%)
Falling onto shoulder	2 (8%)
Falling onto an outstretched arm	3 (11%)
Overhead or throwing sports	2 (8%)
Sudden traction of the arm while lifting a heavy object	17 (65%)
**Follow up**,** mo**	46.8 ± 20.5 (24–94)
**Heaviness of work**	
Light	13 (50%)
Intermediate	8 (31%)
Heavy	5 (19%)

*Significant at *P* ≤.05

M, male; F, female; L, left; R, right; SD, standard deviation

Analysis of the final follow-up PROMs revealed statistically significant improvements in comparison to the preoperative scores (*p* <.001 for VAS, OSS, and SSV) (Table 2). MCID, PASS, and SCB thresholds were calculated, respectively, for OSS (2.8, 7.5, 6.5), SSV (7.5, 32, 25), and VAS (1.2, 3.5, 3.5). Their corresponding sensitivity, specificity, and AUC from the respective ROC curves, are provided in Table 3. Most ROC analyses showed excellent predictive performance (AUC > 0.8), with only one analysis (SCB for VAS) had acceptable (AUC = 78.8) predictive capability (Table 3). The rates of patients achieving MCID, PASS, and SCB were determined, respectively, OSS (96%, 81%, 84%), SSV (100%, 77%, 84%), and VAS (88%, 81%, 81%) (Table 4).The obtained data demonstrate that majority of patients met the clinically significant improvement thresholds at the final follow-up (Table 4).

**Table 2:** Preoperative and postoperative VAS, OSS, and SSV scores

Outcome	Preoperative (mean ± SD)	Final follow-up (mean ± SD)	*P* value *
OSS	32.3 ± 5.7	43.7 ± 5.0	**< 0.001**
SSV	48.4 ± 14.8	90.1 ± 14.0	**< 0.001**
VAS	6.8 ± 2.0	1.6 ± 1.8	**< 0.001**

*Significant at *P* ≤.05. P-values that were statistically significant are presented in bold

SSV, Subjective Shoulder Value; OSS, Oxford Shoulder Score; VAS, Visual Analogue Scale

**Table 3:** MCID, PASS, and SCB threshold values

Variable	Value	Sensitivity (%)	Specificity (%)	AUC (%)
MCID				
OSS	2.8			
SSV	7.5			
VAS	1.2			
**SCB**				
OSS	6.5	95.5	100	97.7
SSV	25	90.9	100	97.7
VAS	3.5	94.4	100	78.8
**PASS**				
OSS	7.5	95.2	80	87.6
SSV	32	90.5	80	83.3
VAS	3.5	95.2	80	85.7

MCID = Minimal Clinically Important Difference; SCB = Substantial Clinical Benefit; PASS = Patient Acceptable Symptom State; VAS = Visual Analog Scale; OSS = Oxford Shoulder Score; SSV = Subjective Shoulder Value

**Table 4:** Patients achieving clinically significant improvements at final Follow-up

Variables	MCID *n* (%)	SCB *n* (%)	PASS *n* (%)
OSS	25 (96%)	22 (84%)	21 (81%)
SSV	26 (100%)	22 (84%)	20 (77%)
VAS	23 (88%)	21 (81%)	21 (81%)

MCID, Minimal Clinically Important Difference; SCB, Substantial Clinical Benefit; PASS, Patient Acceptable Symptom State; VAS, Visual Analog Scale; OSS, Oxford Shoulder Score; SSV, Subjective Shoulder Value

## Discussion

In this study, we aimed to determine the prevalence of concomitant inferior labrum tears in patients with SLAP lesions and assess the clinical outcomes of arthroscopic inferior labrum repair performed alongside SLAP lesion treatment in these patients. The analyses conducted in accordance with these objectives provided several significant findings: (1) inferior labrum tears are a potential concomitant pathology in patients with SLAP lesions, and the majority of these cases are related to forceful traction injuries; (2) in these patients, inferior labrum repair combined with appropriate SLAP treatment achieved successful clinically meaningful outcomes at a mean follow-up of approximately four years.

Traction-related pulling injuries have a significant role in the occurrence of SLAP lesions. Maffet et al. [3] reported that tractional overload, particularly during heavy lifting, is a common etiological factor in this pathology. Similarly, Bey et al. [8] evaluated the effect of inferior humeral translation on the LHBT in a biomechanical study, and demonstrated that cadaveric specimens with inferior humeral translation were more likely to have SLAP lesions. Consistent with these findings, 65% of the patients in the present study had a history of sudden arm traction during heavy lifting, which generated an inferior translational force on the glenohumeral joint. This result supports the association between traction-related injuries and SLAP lesions while emphasizing the importance of obtaining a detailed clinical history of patients.

As observed, the majority of patients had a history of traction-related injury mechanism. This phenomenon could be attributed to the composition of the study cohort, which comprised patients with both SLAP lesions and inferior labrum tears. Limited information is available in the literature regarding injury mechanisms of inferior labrum tears. Page et al. [10] highlighted existing limitations in understanding the occurrence of these injuries, suggesting that a single common mechanism is unlikely. Nonetheless, they stated that the forces causing inferior translation of the humerus could lead to inferior labrum tears. As previously mentioned, the fact that the injury mechanism in most patients in the present study involved forces generating inferior translation supports the proposed view in the literature on the occurrence of inferor labrum tears. In summary, traction-related injuries were the dominant mechanism in patients with concurrent SLAP lesions and inferior labrum tears. This finding again underscores the importance of comprehensive injury history assessment in diagnosing SLAP lesions and suggests that clinicians should evaluate concomitant inferior labrum tears in cases of traction-related injuries.

Another noteworthy aspect of this study is the success of the clinical outcomes achieved through arthroscopic treatment. Studies have demonstrated that patient characteristics and expectations are significant factors in determining the appropriate treatment modalities for SLAP lesions [7, 14, 20–22, 24, 32]. Analyses suggest that repair, tenodesis, or tenotomy procedures, when selected based on these factors, can provide satisfactory clinical outcomes. On the other side, the literature provides limited data regarding the management of inferior labrum tears. Page et al. [10] performed labral repair in 18 patients with inferior labrum tears and reported satisfactory clinical outcomes at a mean follow-up of 44 months. Similarly, Irion et al. [11] observed favorable clinical outcomes and a high rate of return to sports in 12 patients who underwent inferior labrum repair, with a mean follow-up of 37 months. In the present study, the treatment procedure for SLAP lesions was selected with consideration of patient characteristics, and inferior labrum tears were repaired arthroscopically, similar to the studies mentioned above. In the final follow-up results of the present study, with a mean follow-up duration of 47 months, significant improvements in PROMs were observed compared to the preoperative period, supporting the satisfactory clinical outcomes reported in the literature. Additionally, this study assessed clinically meaningful outcomes in patients, including MCID, PASS, and SCB. Cohort-specific analysis within our population revealed excellent or acceptable sensitivity and specificity for the determined thresholds, indicating the reliability of the clinically meaningful outcome assessments. The results demonstrated that satisfactory outcomes were achieved in the study group, not only in terms of numerical data but also from a clinical perspective.

The study also has its limitations. The retrospective nature and reliance on data from a single surgeon’s practice, may have introduced potential biases. The relatively small sample size and the presence of type 2 SLAP lesions in all cases may limit the generalizability of the results and reliable interpretation of the prevalence. In the analysis of patients’ clinical satisfaction, additional variables that could potentially influence treatment outcomes, such as mental health status and preoperative expectations, were not considered. Additionally, the study lacked a comparison group of patients with isolated SLAP lesions, which limits the ability to directly assess the independent effect of inferior labrum tears on clinical outcomes.

## Conclusion

Inferior labrum tears are a pathological condition that may accompany SLAP lesions, and patients with both lesions usually have a history of traction-related injuries. In these cases, successful clinical outcomes could be achieved through patient-specific management of the SLAP lesion and repair of the inferior labrum.

## Data Availability

No datasets were generated or analysed during the current study.
